# The T2238C Human Atrial Natriuretic Peptide Molecular Variant and the Risk of Cardiovascular Diseases

**DOI:** 10.3390/ijms19020540

**Published:** 2018-02-11

**Authors:** Speranza Rubattu, Sebastiano Sciarretta, Simona Marchitti, Franca Bianchi, Maurizio Forte, Massimo Volpe

**Affiliations:** 1 Department of Clinical and Molecular Medicine, School of Medicine and Psychology, Sapienza University of Rome, S. Andrea Hospital, via di Grottarossa 1035, 00189 Rome, Italy; massimo.volpe@uniroma1.it; 2 Istituto di Ricovero e Cura a Carattere Scientifico (IRCCS) Neuromed, Località Camerelle, 86077 Pozzilli (Is), Italy; sebastiano.sciarretta@uniroma1.it (S.S.); simona.marchitti@neuromed.it (S.M.); franca.bianchi@neuromed.it (F.B.); maurizio.forte@neuromed.it (M.F.); 3 Department of Medical-Surgical Sciences and Biotechnologies, Sapienza University of Rome, 04100 Latina, Italy

**Keywords:** atrial natriuretic peptide, T2238C variant, endothelial dysfunction, smooth muscle cells contraction, platelet aggregation, epigenetics, cardiovascular diseases

## Abstract

Atrial natriuretic peptide (ANP) is a cardiac hormone which plays important functions to maintain cardio-renal homeostasis. The peptide structure is highly conserved among species. However, a few gene variants are known to fall within the human ANP gene. The variant rs5065 (T2238C) exerts the most substantial effects. The T to C transition at the 2238 position of the gene (13–23% allele frequency in the general population) leads to the production of a 30-, instead of 28-, amino-acid-long α-carboxy-terminal peptide. In vitro, CC2238/αANP increases the levels of reactive oxygen species and causes endothelial damage, vascular smooth muscle cells contraction, and increased platelet aggregation. These effects are achieved through the deregulated activation of type C natriuretic peptide receptor, the consequent inhibition of adenylate cyclase activity, and the activation of Giα proteins. In vivo, endothelial dysfunction and increased platelet aggregation are present in human subjects carrying the C2238/αANP allele variant. Several studies documented an increased risk of stroke and of myocardial infarction in C2238/αANP carriers. Recently, an incomplete response to antiplatelet therapy in ischemic heart disease patients carrying the C2238/αANP variant and undergoing percutaneous coronary revascularization has been reported. In summary, the overall evidence supports the concept that T2238C/ANP is a cardiovascular genetic risk factor that needs to be taken into account in daily clinical practice.

## 1. Introduction

The atrial natriuretic peptide (ANP) belongs to the natriuretic peptide family along with brain (BNP) and C-type (CNP) natriuretic peptides [1]. It is mainly secreted by the atrial cardiomyocytes and exerts important regulatory functions for the maintenance of cardio-renal homeostasis. The latter is achieved through the activation of guanylyl cyclase (GC)-coupled receptor, mainly GC-A, and an increase of cyclic guanylate monophosphate (cGMP) levels [2]. ANP plays natriuretic, diuretic, and vasorelaxant effects [1,2]. In addition, it exerts antiproliferative, antifibrotic, antiangiogenetic functions and, consequently, it contributes to the cardiovascular remodeling process [3]. An extra-atrial expression of ANP has been reported [4].

The gene encoding ANP (*NPPA*) is located in the distal arm of chromosome 1 (1p36.2), in tandem with the gene encoding brain natriuretic peptide (BNP). It includes three exons and two introns [5]. The signal sequence is located on exon 1, whereas the coding sequence is located on exon 2; exon3 encodes the terminal tyrosine and the 3′ untranslated region. 

Human ANP is synthetized as a pre-prohormone of 151 amino acids and it is subsequently cleaved to obtain a biologically active α-carboxy-terminal peptide along with the amino-terminal end. After removal of the signal peptide, the proANP_1–126_ is released and stored into granules within the atrial cardiomyocytes. Before secretion, proANP_1–126_ is processed by corin, a type II transmembrane serine protease [2], into the circulating forms of ANP_(1–98)_ and ANP_(99–126)_. Of note, the active corin protease is obtained through the cleavage of procorin by PCSK6 [6,7]. The major form of biological active ANP is the 28-amino acid carboxy-terminal peptide, ANP_(99–126)._ On the other hand, further cleavage of the ANP_(1–98)_ generates LANP (long-acting natriuretic peptide), the vessel dilator, and the kaliuretic hormone [8]. The primary structure of αANP is conserved across species, apart from few variations. In particular, an isoleucine is present at position 10 in rats, mice, and rabbits, whereas humans, dogs, and bovines have a methionine at this position. 

Both the high degree of homology and the persistence in the phylogenetic scale support the key role of the ANP primary structure in order to perform its functions. 

The relevance of the ANP primary structure to allow the regular biological activities of the peptide has also been underscored by the discovery of few gene variants that change the amino acid sequence and, consequently, the physiological properties of ANP [9]. 

The first evidence in this regard was obtained in an animal model of spontaneous hypertension and increased predisposition to cerebrovascular disease, the stroke-prone spontaneously hypertensive rat (SHRSP). In this model, the ANP gene was found to map at the peak of linkage of a stroke quantitative trait locus (QTL) [10] and to carry both a variant within the promoter sequence and a variant within the exon 2 sequence, with consequent alterations of peptide regulation, expression, and function [11,12,13]. The latter were associated with the higher stroke susceptibility of the strain [10,11,12,13,14]. 

The subsequent translation of the rat data to the human disease led to very interesting findings. In fact, with regard to the human gene, several variants were reported to fall within the promoter region, the coding, and the intronic parts, as well as within the 3′ end of the gene [9]. These gene variants were associated either with alterations in gene expression and peptide levels (rs5068, G664C) or with an abnormal peptide structure (rs5065). Their contribution to hypertension, coronary artery disease, atrial fibrillation, cardiac hypertrophy, heart failure, and the metabolic syndrome was explored in distinct studies with several remarkable results [9]. 

The present review article will discuss the role of the rs5065 (T2238C) ANP gene variant since it has revealed important biological and pathological effects and has emerged as a relevant risk factor for the development of cardiovascular diseases.

### 1.1. Clinical Evidence of the Pathological Role of the T2238C/ANP Molecular Variant

The T2238C coding variant falls within the exon 3 of the gene and it changes the native stop codon by elongation with two additional amino acids (two arginines). Therefore, the resultant carboxy-terminal peptide contains 30, instead of 28, amino acids.

The frequency of the C allele is about 13–14% in the general population. It has been reported that its frequency may increase up to 23% in populations affected by cardiovascular diseases such as coronary and cerebrovascular diseases [9]. Several case-control association studies were performed in the attempt to explore the pathological relevance of the T2238C transition. As a result, an association of the C2238 allele with an increased risk of both myocardial infarction and stroke was reported in different cohorts, including Caucasian and Asian populations [15,16,17,18,19,20,21,22,23]. Moreover, carriers of this variant allele showed an increased risk of recurrent ischemic stroke and of myocardial infarction [16,18]. Interestingly, an analysis performed in a Caucasian general population found that subjects carrying the C allele had an increased risk to develop stroke and myocardial infarction over a long-term follow up (nine years) [24]. Of note, the C allele carriers had higher BNP values and tended to show an ejection fraction of about 40%, suggesting that their heart may suffer from vascular stress and also from possible direct cardiac consequences (although no evidence of either dilated or hypertrophic cardiac disease was revealed in that study). 

Of note, studies aimed at the identification of a contributory role of T2238C in atrial fibrillation turned out to be negative [25,26]. No clear evidence has ever been obtained with regard to the predisposition to develop hypertension in relation to the carrier status of the C2238 allele. A meta-analysis performed in this regard showed only a modest trend to protect from hypertension [27]. Interestingly, a pharmacogenomic type of approach led to the discovery that hypertensive patients carrying the C2238 allele variant had a greater response to a diuretic-based therapy (chlortalidone) with a significant reduction of their cardiovascular risk [28]. 

Although negative results about the association of the C allele with an increased risk of cardiovascular events were also reported [29,30], the major clinical evidence suggested that the 30- amino-acid-long αANP could exert biological effects different from those of the common αANP, with the production of deleterious consequences for the health status of both coronary and cerebral blood vessels, and with the promotion of lesser diuretic effect. 

Therefore, based on these premises, we decided to pursue detailed molecular studies to identify the precise mechanisms underlying the negative impact of the T2238C/ANP gene variant on the cardiovascular system.

### 1.2. Discovery of the Molecular Mechanisms Underlying the Deleterious Effects of CC2238/αΑnp

In the attempt to fully explore this issue, we undertook a series of in vitro studies in a few vascular cell lines starting with the endothelial cells. Human umbilical vein endothelial cells (HUVEC), once exposed to CC2238/αANP, showed an increased rate of oxidative stress, apoptosis, and necrosis along with reduced cell viability and reduced endothelial cell tube formation [31]. Moreover, CC2238/αANP stimulated the production of proteins involved in atherogenesis [31]. Cannone et al. demonstrated also that CC2238/αANP produced a significant increase of endothelial cell permeability [24]. In this cellular model, the exposure to CC2238/αANP produced a significant decrease of cyclic adenosine monophosphate (cAMP) levels, apart from the expected increase of cyclic guanosine monophosphate (cGMP) levels [32]. In fact, binding affinity studies revealed that CC2238/αANP, compared to the common αANP, has a higher binding affinity for the type C natriuretic peptide receptor (NPR-C) rather than for the type A receptor (NPR-A), and consequently it reduces the adenylate cyclase activity dependent on NPR-C [32]. 

This result represented a remarkable achievement since the NPR-C, devoid of GC activity and mainly known as the clearance receptor of NPs, has direct biological properties that are not fully understood yet [33]. A synthetic form of ANP (C-ANP_4–23_) has been the only known agonist of NPR-C until the discovery of the functional properties of CC2238/αANP [33].

The experimental setup performed in endothelial cells identified a novel mechanism of vascular damage. In fact, the reduction of cAMP levels consequent to the activation of NPR-C, led, in turn, to reduced protein kinase A (PKA) activity and, consequently, to a reduced rate of protein kinase B (Akt) phosphorylation, which is a fundamental factor to maintain endothelial integrity and function [34]. In addition, an increased expression of nicotinamide adenine dinucleotide phosphate (NADPH) oxidase, generating reactive oxygen species (ROS) accumulation, observed in the presence of CC2238/αANP, could certainly contribute to the observed endothelial damage and dysfunction [31,32]. 

To further support our findings, the cAMP reduction upon exposure to CC2238/αANP was abrogated by the inhibition of NPR-C, as well as by the Giα inhibitor pertuxis toxin. More importantly, either the inhibition of NPR-C by antisense oligonucleotide or the selective NPR-C knockdown by RNA gene silencing, rescued CC2238/αANP-induced endothelial cell death [32]. 

Thus, in the pioneering studies performed in endothelial cells, the cAMP-PKA-Akt signalling pathway was revealed as the main one mediating the deleterious effects of CC2238/αANP. Its stimulation appeared to be the consequence of NPR-C rather NPR-A activation, in a manner opposed to that of the regular ANP. The subsequent in vivo translation of these in vitro data, through the characterization of the forearm endothelial-dependent vasorelaxation, revealed that apparently healthy subjects carrying the C2238 allele had a significantly reduced endothelial function [32]. Of note, the reduced endothelial-dependent vasorelaxation was directly related to the number of the mutant alleles, being more severe in double mutant homozygotes. These results provided clear evidence of the clinical implications of the C2238/ANP allele as a hallmark of increased predisposition to human vascular diseases.

A subsequent series of in vitro studies was carried out in both umbilical and coronary smooth muscle cells in the attempt to unravel a potential link between this ANP variant and atherosclerosis [35]. Herein, the in vitro exposure to CC2238/αANP caused oxidative stress damage and increased cell migration and contraction. In this cell line, the role of a reduced cAMP-PKA-Akt axis as well as of the increased NADPH oxidase expression was fully confirmed in response to NPR-C activation. Moreover, we were able to dissect out a novel pathway of vascular damage dependent on PKA inhibition in smooth muscle cells. In fact, we discovered that, as a consequence of PKA inhibition, cAMP response element-binding protein (CREB) was also inhibited, and, further down, microRNA-21 (miR21) expression was reduced, with the expected modulation of miR21 molecular targets. Accordingly, phosphatase and tensin homolog (PTEN) and programmed cell death protein 4 (PDCD4) increased, whereas B-cell lymphoma 2 (Bcl2) decreased. As a result of the modulation of these deleterious mechanisms, a severely compromised vascular smooth muscle cell viability was observed in the presence of CC2238/αANP [35]. Thus, our set of data provided an original evidence for the existence of an epigenetic regulation mediating the deleterious effects of CC2238/αANP in vascular cells. The replacement of miR21 could restore smooth muscle cell viability and function in the presence of CC2238/αANP.

miR21 is known as a fundamental protective factor for maintaining viability and proliferation in several cell types and it has been found to be highly expressed in all main types of cardiovascular cells [36]. It plays important roles in vascular smooth muscle cell proliferation and apoptosis through the targeting of PTEN, Bcl2, and PDCD4 proteins [37,38,39]. Notably, the antiproliferative effect of wild type αANP has been previously shown to associate with a decrease of miR21 in aortic smooth muscle cells, whereas the overexpression of miR21 restored vascular smooth muscle cell proliferation [40]. In addition, our studies supported previous evidence that miR21 belongs to the Akt regulatory network in cardiovascular cells as an upstream factor, because of its ability to inhibit PTEN and to exert, through the consequent increased Akt activity, important effects on cell proliferation [37,41]. In fact, both PTEN and Akt represent key molecules for cell growth and survival, as well as for the development of many cardiovascular diseases [42]. Finally, these original results were able to support knowledge on the ability of the cAMP-PKA-CREB axis to regulate the expression of miRNAs in vascular cells [43,44].

The relevance of an epigenetic regulation underlying some of the vascular effects of CC2238/αANP was later on reinforced by additional findings on the role of microRNA-199a (miR199a) in the same cell line [45]. In fact, we searched for differential gene expression of atherosclerosis-related pathways in vascular smooth muscle cells exposed to either TT2238/αANP (wild type) or to the variant CC2238/αANP. The major finding was that CC2238/αANP induced Apolipoprotein E (ApoE) downregulation through NPR-C-dependent mechanisms involving the upregulation of miR199a-3p and miR199a-5p and the downregulation of DnaJ (Hsp40) homolog (DNAJA4). The reduced expression of ApoE by CC2238/αANP was associated with a significant increase of inflammation, apoptosis, and necrosis that were completely rescued by the exogenous administration of recombinant ApoE [45]. Of note, the upregulation of miR199a by NPR-C was mediated by a ROS-dependent increase of early growth response protein-1 (Egr-1) transcription factor. In fact, Egr-1 knockdown abolished the impact of CC2238/αANP on ApoE and miR199a. As expected, NPR-C knockdown rescued the ApoE levels [45]. 

This set of in vitro data provided the first original demonstration that CC2238/αANP, through NPR-C-dependent activation of Egr-1 and the consequent upregulation of miR199a, downregulates ApoE in vascular smooth muscle cells. ApoE is a 34 kDa protein that participates in the mobilization and distribution of cholesterol and of other lipids among various tissues of the body [46]. ApoE was previously found to be expressed in vascular smooth muscle cells where ApoE downregulation is positively correlated with typical markers of inflammation, such as small mother against decapentaplegic 4 (Smad4) [47] and nuclear factor kappa-light-chain-enhancer of activated B cells (NF-κB) [48]. Previous studies demonstrated that ApoE exerts also pleiotropic anti-atherosclerotic and anti-inflammatory cellular effects. It is known that ApoE deficiency alone promotes the development of aortic atherosclerotic plaques in mice [49]. In humans, where the lack of ApoE is rare, the risk of atherosclerosis is strongly associated with three common apoE isoforms in the order of APOE4>APOE3>APOE2 [46]. Furthermore, ApoE is known to play a role in the development of cardiovascular diseases [50]. Thus, our studies may suggest that CC2238/αANP induces vascular damage also through the inhibition of the above-described pleiotropic effects of ApoE. As a consequence, restoring ApoE levels could represent a potential therapeutic strategy to counteract the harmful effects of CC2238/αANP in subjects carriers of the variant peptide.

A third cellular element stimulated our interest, on the basis of the knowledge that platelets play a key role in the promotion of atherothrombotic events [51] and on previous evidence of a certain functional impact of ANP on platelet aggregation [52]. The hypothesis was that CC2238/αANP could exert pro-aggregant effects on platelets and therefore, through this mechanism, could also promote the higher occurrence of stroke and myocardial infarction in human subjects.

In fact, we discovered that CC2238/αANP favored platelet activation and aggregation in vitro through the activation of NPR-C and the reduction of cAMP levels [53]. It increased ROS production through the upregulation of Nox2, a major NADPH oxidase isoform that is abundant in platelets [54]. More importantly, a translation of the in vitro data to the human condition was achieved through the demonstration that patients with atrial fibrillation (who are more prone to thromboembolic events) had a higher level of platelet aggregation and activation and of oxidative stress if they were also carriers of the C2238/αANP allele [53]. On the basis of these observations, Nox2 inhibition may represent a potential intervention to decrease the cardiovascular risk in patients carrying the C2238/αANP allele. These findings were subsequently extended to ischemic heart disease with a study performed in patients undergoing percutaneous coronary revascularization and taking dual antiplatelet therapy. The study revealed that carriers of the C2238/ANP allele had a higher residual platelet reactivity when diabetic [55]. Since diabetes represents on its own a pathological condition characterized by an increased platelet aggregation [56], a close monitoring should be deserved to this cohort of patients undergoing coronary revascularization if they are carriers of the T2238C/ANP variant.

Altogether, the combined evidence of endothelial damage and dysfunction, of increased smooth muscle cells migration and contraction with decreased levels of the protective ApoE, and of increased platelet activation and aggregation outline a pathway that may favor atherosclerotic plaque formation as well as plaque instability up to thrombus formation, with the consequent cardiovascular acute event. 

Obviously, CC2238/αANP should be interpreted as one of the factors contributing to the above-mentioned deleterious effects on the vessel wall and to the consequent clinical outcome.

## 2. Summary and Outlook

**Figure 1:**
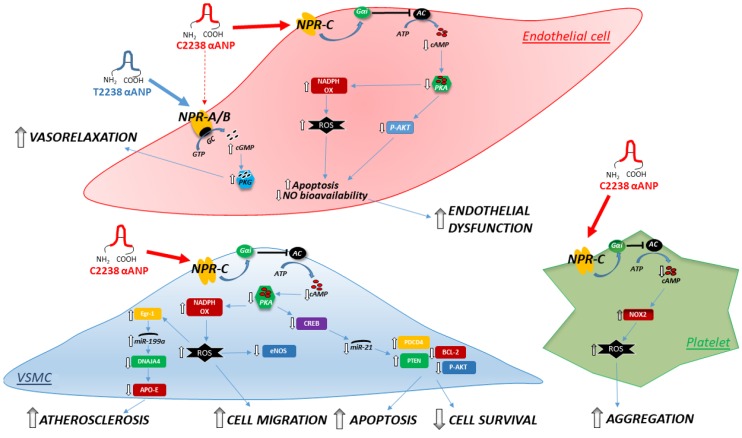
Schematic representation of the molecular signaling pathways underlying the effects of CC2238/αANP in endothelial cells, smooth muscle cells, and platelets. Abbreviations: AC, adenylate cyclase; Akt, protein kinase B; APO-E, apolipoprotein E; ATP, adenosine triphosphate; BCL-2, B-cell lymphoma 2; cAMP, cyclic adenosine monophosphate; cGMP, cyclic guanosine monophosphate; CREB, cAMP response element-binding protein; DNAJA4, DnaJ (Hsp40) homolog; Egr-1, early growth response protein-1; eNOS, endothelial nitric oxide synthase; GC, guanylate cyclase; GTP, guanosine triphosphate; Gαi, inhibitory G protein; miR-21, microRNA-21; miR-199, microRNA-199; NADPH OX, nicotinamide adenine dinucleotide phosphate-oxidase; NO, nitric oxide; NOX2, NADPH oxidase 2; NPR-A/B, natriuretic peptide type A/B receptors; NPR-C, natriuretic peptide type C receptor; P-AKT, phosphoprotein kinase B; PDCD4, programmed cell death protein 4; PKA, protein kinase A; PKG, protein kinase G; PTEN, phosphatase and tensin homolog; ROS, reactive oxygen species; VSMCs, vascular smooth muscle cells; αANP, carboxy-terminal atrial natriuretic peptide, wild-type (T2238) and mutant (C2238). Arrows indicate the positive stimulation within each pathway. The dotted arrow indicates lower affinity binding of C2238 for NPRA/B.

Dissecting out novel pathways of vascular damage dependent on a gene variant is an intriguing issue from both a scientific and a clinical point of view. In the case of T2238C/ANP, we demonstrated that the extended (30-amino acid) gene product acts through the activation of a specific receptor (the NPR-C receptor) to induce multiple deleterious mechanisms. In addition, the ANP variant targets several cell types belonging to both the vascular wall and the bloodstream and, by doing so, it increases the cardiovascular risk (Figure 1).

Interestingly, the findings describe for the first time the cAMP-PKA axis as the one driving all other signaling events stemming from CC2238/αANP, such as the CREB-miR21and the Egr-1-miR199a-ApoE pathways in smooth muscle cells, NADPH increase, and particularly Nox2 increase in platelets (Figure 1). In vivo, we obtained evidence of reduced endothelial-dependent vasorelaxation in apparently healthy subjects, of increased platelet activation and aggregation in a cohort of patients with atrial fibrillation, and of higher residual platelet aggregation in ischemic heart disease diabetic patients (following administration of dual antiplatelet therapy) if they were carrier of the C2238/αANP variant [55]. A previous pharmacogenomic study had already revealed the greater efficacy of diuretic therapy in hypertensive carriers of the C2238/ANP variant allele [28].

The findings provide original evidence in the field of the pathogenesis of vascular damage. We believe that the complex, multifaceted mechanisms explaining the negative impact of a molecular variant of ANP give even more support to the beneficial role played by the common form of ANP within the cardiovascular system. In fact, we can better support the view that the common form of ANP, acting through NPR-A activation and the consequent cGMP release, is a peptide promoting vascular health, angiogenesis, vascular smooth muscle cells relaxation, regular platelets activation and aggregation. On the other hand, the change in the peptide sequence leads to higher binding affinity for NPR-C and introduces several differences in the functions of the peptide, making C2238/ANP a “toxic” substance for the vascular wall and for the cardiovascular system generally [57]. 

A few final considerations are needed in regard to what we have learned about the T2238C/ANP variant. First of all, discovering a functional biological effect of a gene variant is not common. Furthermore, the translation of in vitro effects to in vivo condition is even less likely. Even when this achievement is obtained, the expected effect in vivo is usually small in the context of complex multifactorial diseases. 

The case of CC2238/αANP is uncommon. In fact, apart from dissecting out its specific biological effects, the molecular studies allowed the discovery of a novel role of a known receptor (NPR-C) and of novel signaling pathways leading to vascular damage. More importantly, the deleterious impact of the alternative peptide can be detected in vivo, which is uncommon and likely to be of significant relevance to the physiological effects of ANP.

Therefore, the current mechanistic knowledge on C2238/αANP underscores a novel cardiovascular genetic risk factor that needs to be taken into account in daily clinical practice. Of note, the cost of the determination of the presence of the T2238C allele is affordable.

Further studies should assess whether T2238C allele characterization may add some value on top of the conventional risk factors and may have clinical and therapeutic relevance. 

Moreover, some of the molecules outlined in our studies could be targeted by novel therapeutic approaches to reduce the cardiovascular risk in carriers of the C2238/ANP allele.
